# Protocol: optimising hydroponic growth systems for nutritional and physiological analysis of *Arabidopsis thaliana* and other plants

**DOI:** 10.1186/1746-4811-9-4

**Published:** 2013-02-05

**Authors:** Simon J Conn, Bradleigh Hocking, Maclin Dayod, Bo Xu, Asmini Athman, Sam Henderson, Lucy Aukett, Vanessa Conn, Monique K Shearer, Sigfredo Fuentes, Stephen D Tyerman, Matthew Gilliham

**Affiliations:** 1 School of Agriculture, Food & Wine and The Waite Research Institute, University of Adelaide Waite Campus, PMB1, Glen Osmond, South Australia 5064, Australia; 2 Australian Research Council Centre of Excellence in Plant Energy Biology, Glen Osmond, South Australia 5064, Australia; 3 Australian Centre for Plant Functional Genomics, Glen Osmond, South Australia 5064, Australia

**Keywords:** Hydroponics, Plant nutrition, Arabidopsis, Gas exchange, ACA2, CAX1, CAX2, VHA-α, Transient transformation

## Abstract

**Background:**

Hydroponic growth systems are a convenient platform for studying whole plant physiology. However, we found through trialling systems as they are described in the literature that our experiments were frequently confounded by factors that affected plant growth, including algal contamination and hypoxia. We also found the way in which the plants were grown made them poorly amenable to a number of common physiological assays.

**Results:**

The drivers for the development of this hydroponic system were: 1) the exclusion of light from the growth solution; 2) to simplify the handling of individual plants, and 3) the growth of the plant to allow easy implementation of multiple assays. These aims were all met by the use of pierced lids of black microcentrifuge tubes. Seed was germinated on a lid filled with an agar-containing germination media immersed in the same solution. Following germination, the liquid growth media was exchanged with the experimental solution, and after 14-21 days seedlings were transferred to larger tanks with aerated solution where they remained until experimentation. We provide details of the protocol including composition of the basal growth solution, and separate solutions with altered calcium, magnesium, potassium or sodium supply whilst maintaining the activity of the majority of other ions. We demonstrate the adaptability of this system for: gas exchange measurement on single leaves and whole plants; qRT-PCR to probe the transcriptional response of roots or shoots to altered nutrient composition in the growth solution (we demonstrate this using high and low calcium supply); producing highly competent mesophyll protoplasts; and, accelerating the screening of Arabidopsis transformants. This system is also ideal for manipulating plants for micropipette techniques such as electrophysiology or SiCSA.

**Conclusions:**

We present an optimised plant hydroponic culture system that can be quickly and cheaply constructed, and produces plants with similar growth kinetics to soil-grown plants, but with the advantage of being a versatile platform for a myriad of physiological and molecular biological measurements on all plant tissues at all developmental stages. We present ‘tips and tricks’ for the easy adoption of this hydroponic culture system.

## Introduction

*Arabidopsis thaliana* (L.) Heynh. (Arabidopsis) has been adopted as a model plant of choice in many laboratories for a variety of reasons. These include a brief life cycle, a small and well-annotated genome, its amenability to tissue culture, the limited cell-layers per cell type (for developing roots), the availability of natural diversity sets and targeted mutants, and the ease at which it can be genetically transformed [1]. The diminutive stature and rosette growth habit of Arabidopsis also means that it does not require a large area to cultivate. At the same time, the size of Arabidopsis has presented considerable challenges for those wanting to perform physiological measurements on intact plants such as gas exchange, hydraulic conductance, or for obtaining single-cell parameters such as turgor pressure and membrane potential. To benefit from the vast molecular resources of Arabidopsis, physiologists have had to adapt measuring equipment and assays to the microscale; these technological challenges have curtailed the use of Arabidopsis as a tractable physiological model [2]. In order to perform such assays whilst providing a flexible experimental platform for manipulation of both the shoot and root environment, the use of hydroponics for research purposes has become common.

Hydroponics, as a convenient means for studying plants in the laboratory and for growing commercial crops, was a term first coined by William F. Gericke in 1929, yet it is a documented technique dating back to the late 17^th^ century [3,4]. Its advantages include the potential for accessibility to all plant tissues and the easy manipulation of the nutrient profile of the growth medium when compared to soil, given the complex interaction of ions with soil particles. Agar or phytagel plates share these advantages but the opportunities for physiological experimentation using this system are limited as seedlings can only be grown for about 2 weeks on plates and plants transpire very little meaning that sucrose is commonly included as a carbon substrate and aseptic culture must be used. A disadvantage of both hydroponics and agar plates is that many species have a different root morphology when compared to soil, including a lack of root hairs, although this is not the case for Arabidopsis [5]. Various hydroponic systems have been developed for the growth of Arabidopsis independently in several laboratories reflecting their need and utility; Araponics^©^, is an example of a commercially available system [6]. Other hydroponics systems described in the literature have often been designed with a specific purpose in mind, and as a result have not been tested in terms of the ease at which various experimental parameters can be assayed (refer to Table 1 for advantages and disadvantages of each method). Whilst trialling these methods in our laboratory we identified several key limitations with these systems as they are documented in the literature, including: (1) the use of a small holding tank (up to 1 L) to hold the growth solution, reducing scalability [7,8]; (2) the need to sterilise parts of the set-up [8,9], which lengthened and complicated the procedure; (3) the use of rockwool or sponge [7,10-12], which prevented access to the full root system and predisposed the apical meristems to flooding; (4) the use of specialised materials such as a prefabricated seed holder, which increased cost [13]; and the need to transfer plantlets between multiple growth environments [14,15]. While each methodology possessed strengths and was designed to suit its endpoint analysis, we sought to streamline the entire process and provide a universal and fully adaptable system.

**Table 1:** Advantages and disadvantages between geoponics, agar plates and three distinct aggregate hydroponics methods for cultivating arabidopsis plants

**Parameter**	**Geoponics (i.e. soil/sand)**	**Agar plates**	**Aggregate hydroponics**		
			**Polystyrene/ Rockwool**	**Araponics©**	**This system (Conn *et al.*)**
**Setup costs**	Low	Low	Low	High	Low
**Running costs**					
**Media**	Low	Intermediate	Low	Intermediate	Low
**Equipment**	Low	Intermediate	Low	Intermediate	Low
**Footprint**	Small	Small	Small	Small-to- Intermediate*	Small
**Sterile culture**	No	Yes	No	No	No
**Batch variability**	High	Low	Low	Low	Low
**Experimental Flexibility**	Low	Intermediate	High	High	High
**Contamination (algal, bacterial)**	Medium	High	High	Low	Low
**Throughput**	High	Intermediate	Intermediate	Intermediate	Intermediate
**Root entanglement**	Yes	Potential	Yes	Yes	No
**Developmental window**	Mature plants	< 3 week-old seedlings	Mature plants	Mature plants	Mature plants

* Can use either the low or high density trays.

The system presented in this manuscript is regarded as agar-based, aggregate hydroponics. Estimated setup costs for Araponics system is approximately US$4 per plant, while for the system presented in this paper is US$0.81 (based on 140 plants, pricing as per January 2013, http://www.araponics.com).

One common and significant problem associated with aggregate hydroponics growth systems is the algal contamination of the culture medium [16]. This can occur in the tank, and particularly on rockwool or agar-based plugs, or the plant roots and shoots, due to the use of non-sterile phosphorous-rich medium and the exposure of these components to light. This becomes a problem for physiological studies as algal growth can reduce root nutrient uptake efficiency, plant growth, perturb the composition of the growth solution (nutrients, pH) and induce significant changes to the plant global transcriptome and proteome [16-18]. For this reason alone it is important that hydroponic systems avoid illumination of the growth media if they are to be used in physiological studies.

A major driver for developing this hydroponics system was to be able to manipulate Arabidopsis plants for a variety of assays including single cell sampling and analysis (SiCSA), which requires live plants to be fixed to a flat, hard growth surface [19-22]. The following system allowed us to sample single cells easily for both molecular and ionomic interrogation (the methodology for molecular analysis is outlined in [22]); it would also be ideal for micropipette techniques such as turgor measurement and electrophysiology. After considerable iterative development we present this simple, inexpensive, flexible and robust hydroponics system for the cultivation of Arabidopsis (and other plants), which addresses the above considerations and streamlines the methodology to allow other laboratories to adopt this procedure. In addition, we document how to adapt physiological measuring equipment to this hydroponics system and present some analyses of Arabidopsis plants grown in this hydroponic set-up. These comparisons show that the hydroponic system produces plants with equivalent growth rates to soil-grown plants but provides more flexibility for applying many physiological and molecular analyses of the plant tissues.

## Materials

### Reagents

• Agar, plant cell culture tested (e.g. Sigma, A7921)

• Nutrient solution stocks (see Additional file 1 for detailed description of growth solutions).

### Equipment

• 1.5 mL microcentrifuge tube, black(e.g. Bioplastics, B74010), 48

• 50 mL polypropylene conical centrifuge tube with flat top screw cap (e.g. BD Biosciences, 352070), 48

• Leather punch, or 15-18G × 1 1/2" hypodermic needles (e.g. Terumo, NN-1838R), 1

• 13 L multistacking container (e.g. Nally, IH305), 1

• 24 well floater microtube rack, blue with hinged lid (Scientific Specialties, 5100-43), 2

• Or, for large scale planting ( > 100 seeds) pizza crisper trays with 11 mm holes (e.g. Willow, heavy metal bakeware 34 cm family size) and pot saucer that fits the pizza tray making it light tight (e.g. Reko, 430 mm saucer, RSRSTD430.07), 1 each.

• Plastic support for tubes in hydroponics container, plastic, 1

• Aquarium air pump (e.g. Resun, AC9904), 1

• Freshwater aquarium air stones, 2

• Aquarium tubing, 1.5 m to fit aquarium pump (e.g. Aquaone, 4 mm internal diameter tube)

• Plastic Y-connectors to fit tubing, and clamps to adjust airflow

• Fluorescent lamps 36W/840 cool white (e.g. Osram, 4050300517872).

### Equipment setup

#### Mature plant tank

• Remove the conical base from the 50 mL centrifuge tubes using a hacksaw or band saw, and smooth the cut edges with a metal file to prevent future root damage. Drill a hole in the centre of 50 mL centrifuge tube lid (11 mm diameter) to support the lip of the plant holder. Forty-eight tubes are required per tank.

• Adhere four plastic strips (20 × 120 × 10 mm) to the inside of an opaque 13 L hydroponics growth container (320 mm × 415 mm × 110 mm) with silicon-based adhesive, 20 mm from the top to support the microcentrifuge tube lid.

• Plastic lids can be made from a rectangular plastic sheet (290 mm × 390 mm × 5 mm). Using a hole-bit drill 48 holes (6 × 8 pattern) of 32 mm diameter to fit the cut 50 mL centrifuge tubes.

• Aeration of each hydroponics tank is provided via a single tube from a 4-outlet aquarium pump (5 W, 540 L.h^-1^ maximum), with a Y-connector fitted inline to permit the use of two freshwater airstones (30 –100 mm) in each tank. These can be anchored onto the base of the tank with silicone adhesive. Use clamps to adjust airflow if necessary.

• Plants in hydroponics tanks can be illuminated as required. For this setup we use 36W/840 cool white fluorescent lamps, 8 lamps per shelf (3 tanks per shelf). Plants are typically grown 210 mm beneath lamps.

### Protocol

**Figure 1:**
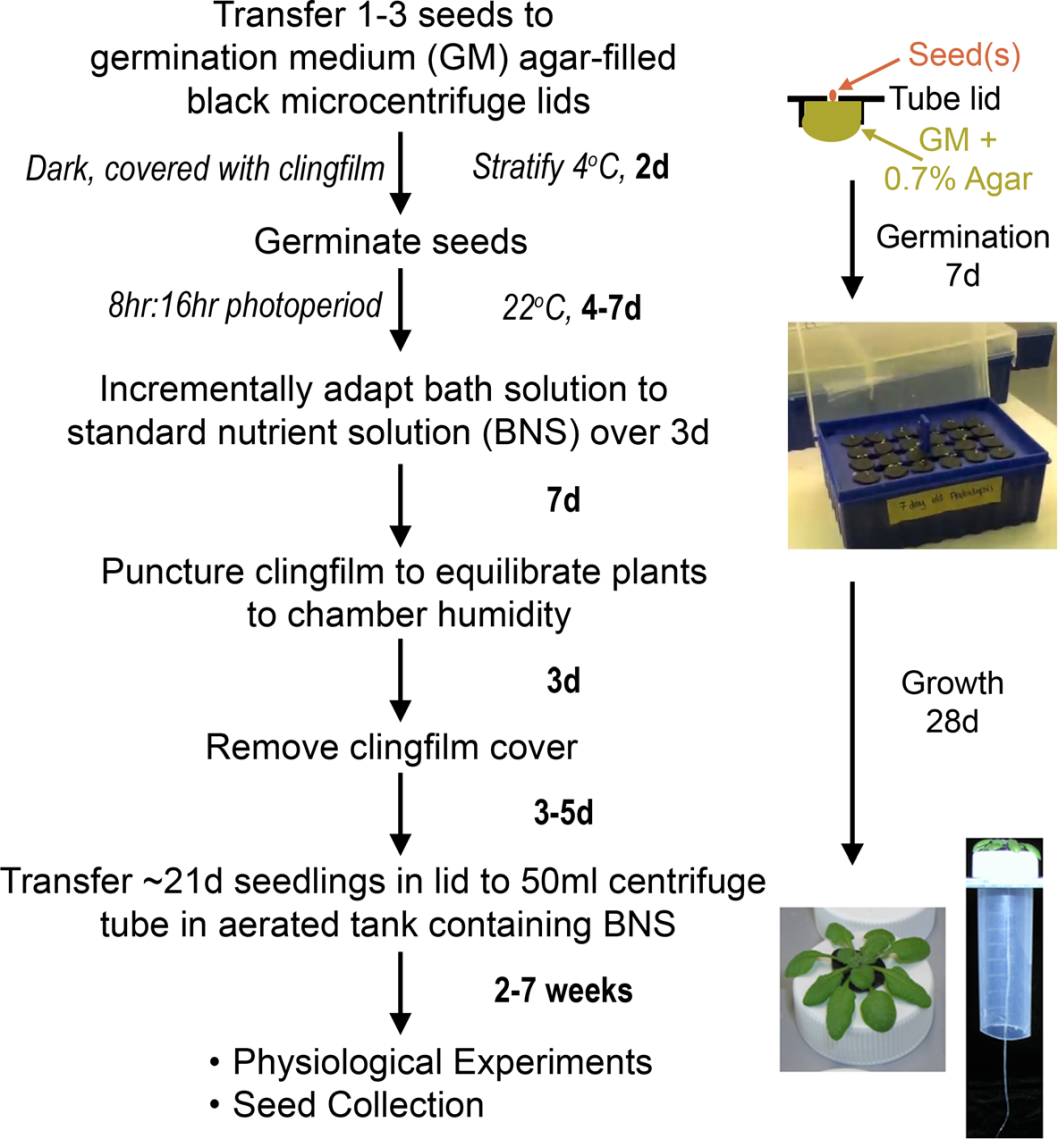
**Simplified Arabidopsis hydroponics growth method.** Flow chart outlining the timeline and key steps in the process. Timing (in bold) on right of arrows indicate time between steps (d: days). Images on right-hand panel showing setup of seed germination and representative images of seedlings and mature plants, including view of roots contained within centrifuge tubes of 5-week old plant. Also refer to protocol, and Additional file 2 and online tutorial video (http://www.youtube.com/watch?v=c9neVLaS63c) for more detailed descriptions of the equipment set-up.

The general workflow for the Arabidopsis hydroponics system is summarised in Figure 1, Additional File 2, with step-wise written instructions below and is further outlined in a tutorial video (http://www.youtube.com/watch?v=c9neVLaS63c). The total cost to completely establish this system, at current prices, is up to five times less than commercially sourced systems. The ability to reuse the majority of the components further reduces the expense of the system.

### Preparing germination lids

1. Prepare 100 mL of germination medium (GM) (recipe Additional file 1) in a autoclavable bottle and add 0.7 g agar (final conc. 0.7% w/v), autoclave and cool slightly. The solution can also be microwaved to dissolve the agar as aseptic culture is not required.

2. Using a leather punch or hypodermic needle, bore a single 1.2 – 1.8mm diameter hole into the centre of a black microcentrifuge tube lid.

*NOTE*: This design is essential to limit light penetration into the culture medium and in so doing abolishes algal growth and minimises evaporation/water loss from the hydroponic tanks.

3. Cut lids from the microcentrifuge tube base, retaining 1 – 2mm of the hinge, invert lid onto clingfilm or adhesive tape. Once all lids have been prepared, fill each with ~250-300 μL germination medium agar and leave to solidify for 15 min.

*CRITICAL POINT:* The hinge of the microcentrifuge lid can be used for easy manipulation of plants with tweezers.

*CRITICAL POINT*: Ensure the lids are filled such that there is a dome of GM-agar for each lid, but avoid overflowing as this may cause the lids to sit askew in the germination tank. If the solution escapes through the lid hole, either allow media to cool (55-60°C is ideal) or supplement with additional media.

*NOTE*: Once finished, the residual GM-agar can be stored at 4°C for 1 month and reused by melting in the microwave as required.

4. Invert lids into floating racks with the agar plug in contact with liquid GM to create the functional seed/seedling/plant holder.

*NOTE*: Prefabricated 34 cm diameter pizza crisper trays, containing over three hundred holes of 11 mm in diameter, can be used to hold larger batches of plants. We found it essential that each of the microcentrifuge tube lids sat snugly enough in the holding tray to prevent light penetration into the GM, but loosely enough so they could be easily removed and transferred to another container when required.

### Germinating seedlings

5. Using a moistened toothpick, place up to three seeds in the hole of the lid on the agar surface to maximise chances of seed germination. Then, cover the entire container with plastic clingfilm to enhance humidity, leaving at least 10 mm above the plant for growth. Stratify seeds in the dark at 4°C for at least 48 h.

6. Transfer the germination tank into growth cabinets under a 8:16 h, light:dark cycle, with 55% atmospheric humidity, at 22°C and an irradiance of 150 μmol photons m^-2^.s^-1^ at the plant level. Under these conditions, the roots of these seedlings emerge from the agar plug after 4-7 days.

7. At this stage thin down to a single plant per lid and replace the bath solution incrementally from GM to a standard growth solution (in our case, a modified ¼ Hoagland's solution, hereafter referred to as BNS, refer to Additional file 1 for recipe). On day 1 of the solution change, ^1^/_3_ of the GM was replaced with BNS. On day 2, 50% of the existing solution was exchanged with BNS and on day three the entire solution was replaced with BNS.

8. After day 14, puncture holes in the clingfilm to decrease humidity and then remove completely after day 17.

*CRITICAL POINT:* Do not allow agar plugs to dry out at this stage, this is rarely a problem if using floating racks but it is extremely important to keep the solution level topped up if using pizza or equivalent trays to germinate the seedlings.

### Maturing plants

9. When the roots are 40–50 mm in length, approximately 21 days post-germination, plants are the appropriate size to survive transfer into an aerated hydroponics tank. Transfer plants in lids to the modified 50 mL centrifuge tubes, passing the roots through the 11 mm diameter hole drilled in its lid to support the lip of the seedling holder. Then insert this unit into the lid of the tank containing 10 L of growth solution and continue until all 48 positions are filled.

*CRITICAL POINT*: These holders permit access for the roots to the whole growth media but prevent root entanglement for up to ~7 weeks when grown under short (8 h:16h) photoperiod (Figure 1).

*NOTE*: If not all 48 plant tubes are filled with plants, unused holes must be covered to exclude light from the growth solution. Use 50 mL centrifuge tube lids without holes or place an intact lid or base of a black microcentrifuge tube within the 11 mm hole if present, or use large pieces of aluminium foil wrapped in plastic clingfilm to cover multiple holes simultaneously.

*NOTE*: Plants can remain in these 13 L tanks, each holding 48 plants, with weekly solution changes until analysis. After ~3 days in these larger tanks the agar plug dries to form a thin film that separates itself from the root system. This occurs because the agar plug no longer is in contact with the growth solution when the plant holder is placed in the hydroponic tank. As such, this permits full access to the whole of the shoot and/or root system. The plant holder provides a useful handling tool for transferring the seedling to experimental chambers or different solutions, whilst limiting mechanical stress, but could be removed from the plant by cutting the plastic lid in half. This is particularly useful for imaging whole plants for reporter localisation studies.

### Sample results

#### Plant growth and seed collection

**Figure 2:**
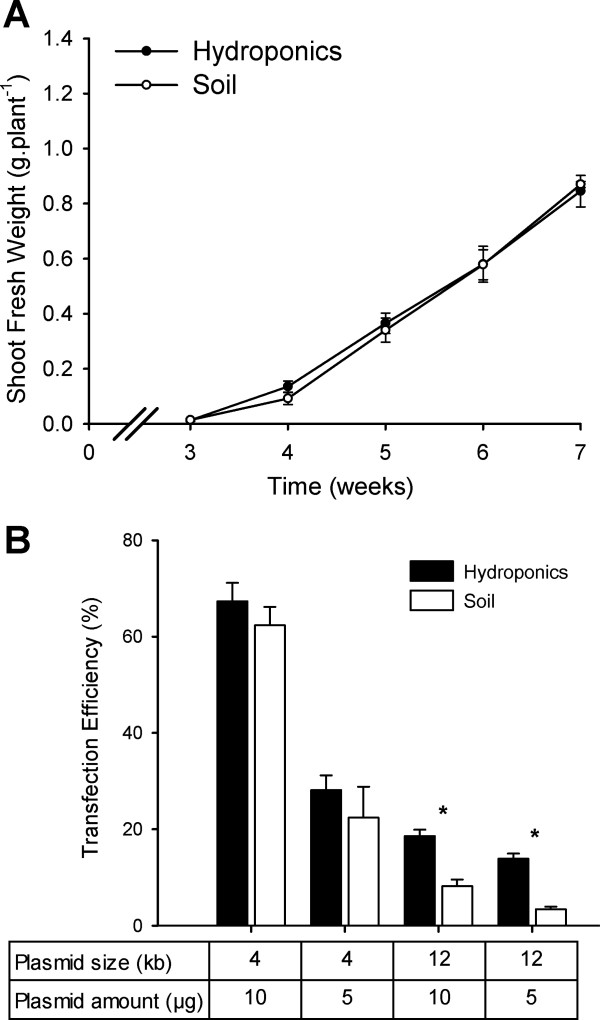
**Comparisons of Arabidopsis shoot growth kinetics and protoplast transformation efficiency between soil and hydroponics system. A**) Shoot biomass during vegetative growth phase of Arabidopsis Col-0 is equivalent between soil-grown and hydroponically-grown plants under short-day photoperiod (8 h:16 h) until seven weeks post-germination. Mean ± SD (n = 6 plants per timepoint, per condition). No significant differences were found between growth conditions at each timepoint using a *t*-test (P < 0.01). **B**) Transfection efficiency of Arabidopsis mesophyll protoplasts were determined by fluorescence microscopy comparing two quantities (5 μg and 10 μg) of two sGFP-expressing plasmids under a single CaMV 35S promoter, pHBT-sGFP(S65T)-NOS (GenBank accession number: EF090408) [25] and pGWB406 (GenBank accession number: AB294430) [37] of 4.2 kb and 12.4 kb, respectively as per Conn *et al. *[20]*.* For each condition n = 5 independent transformations, each with cell counts > 100 protoplasts. Data presented as the proportion of GFP-expressing cells; Mean + SEM. Asterisks indicate significant difference between soil and hydroponics derived protoplasts within each condition (P < 0.01).

Plant growth and development are dynamic processes that can be perturbed by a number of biotic and abiotic factors, including nutrient availability, oxygenation of growth solutions, prevalence of microorganisms, humidity and air temperature [23]. A number of measurements were made to ascertain the physiological state of plants grown in our hydroponics system. Under both soil and hydroponic conditions using the BNS growth solution, plants had vibrant green colouration (total chlorophyll content of 5-week old hydroponics plant leaves was 12.5 ± 0.4 μg.mg-DW^-1^ (mean ± SD) while soil-grown leaves had 12.6 ± 0.6 μg.mg-DW^-1^ (n = 12), both with approximately 2.5:1 of Chl*a*:Chl*b*), and possessed the same growth rates throughout the vegetative growth cycle (3-7 weeks) (Figure 2A). The germination rate of plants grown hydroponically was 5–18% higher than on soil (supplemented with seed raising mix for 20 lines tested), with the greatest increase seen for the *cax1-1*/*cax3-1* T-DNA insertion line [24]. Once siliques were filled, plants were wrapped in clear, perforated plastic bags and transferred into tanks containing ~2 L water to avoid salt formation on roots, to avoid mould growth and to hasten drying. Siliques dried upon evaporation of the water, with the isolated seeds possessing from 90–100% germination efficiency and unaffected leaf ionomics profile compared to the previous generations (data not shown).

### Transient protoplast transformation

A number of studies on promoter responsiveness, cellular localisation and protein-protein interactions can be undertaken in Arabidopsis protoplasts, rather than using the whole plants. Yoo *et al. *[25] presented a technique for transient expression of genes in protoplasts isolated from Arabidopsis mesophyll cells. We trialled a modified version of this protocol on protoplasts isolated from plants grown in soil or our hydroponics system, to detect expression of a cytosolic *sGFP* encoded on both a small vector (4 kb) and a large vector (12 kb), and quantifying the proportion of GFP-positive cells. The transfection efficiency of protoplasts derived from hydroponically-grown leaves was consistently higher than that of those derived from soil-grown plants, at least 2-fold higher for the 12 kb vector and 8–26% higher for the smaller vector, depending on DNA input (Figure 2B). Furthermore, as expected, we observed that the transformation efficiency of the larger vector was lower regardless of growth regime. However, for the hydroponically grown plants at least, the rate was sufficiently high at 14–20% to be used as a screening tool for specific applications like subcellular localisation of large membrane transporters. No difference was observed in the average size of protoplasts between methods, or the intracellular localisation of sGFP, yet the total yield of mesophyll protoplasts was consistently 15% above those from soil-grown plants, in part due to more uniform growth enabling the harvest of healthy leaves at consistent stages of development. Combining this higher yield and higher transformation rate, this constitutes an optimised approach for the study of many processes in protoplasts.

### Plant nutrition and transcriptional response

We tested a number of plant growth solutions and found that a modified ¼ Hoagland’s solution (BNS) was a simple, defined and affordable media, which supported good plant growth and similar nutrition to plants grown in soil obtained from the largest public dataset for Arabidopsis ionomics, the PiiMS database (http://www.ionomicshub.org) (Table 2). Note that the PiiMS soil-grown plants were fed ½-strength Murashige and Skoog (MS) medium. As a result of previously observing growth retardation and stress phenotypes associated with full strength growth solution (i.e. Gamborg's, Hoagland’s or MS) we used the more dilute BNS media, as it was sufficient to provide adequate and reproducible growth. The flexibility afforded by creating the growth solution from individual components allowed manipulation of certain nutrients either separately or in combination in order to investigate nutrition-associated genotypes or phenotypes of different Arabidopsis ecotypes or mutant lines [19-21]. The basic recipe plus those with altered (increased or decreased) concentrations of potassium (K), sodium (Na), calcium (Ca) [19,21] and magnesium (Mg) [20] can be found in Additional file 1. In each of these solutions the concentrations of multiple components were altered to keep the activity of most ions the same despite a significant change in the one or two of the ion species. This was performed using the ion activity calculator program vMinteq (KTH) to investigate, as far as possible, ion-specific treatments [19-21].

**Table 2:** Comparative ionomics of soil-grown and hydroponically-grown plants

**Element**	**Soil-grown**	**Hydroponics**	**Ratio**
Na^23^	1,608 ± 219	1,808 ± 120	1.12
Mg^25^	9,402 ± 845	9,876 ± 492	1.05
P^31^	8,449 ± 602	8,225 ± 204	0.97
K^39^	34,214 ± 1874	37,747 ± 1542	1.11
Ca^43^	44,314 ± 3005	38,821 ± 1603	0.86
**Micronutrients**			
Cr^52^	0	< 4	n.d.
Mn^55^	64 ± 35	116 ± 42	1.82
Fe^56^	85 ± 36	64 ± 20	0.75
Co^59^	3 ± 0.6	< 6	n.d.
Ni^60^	1 ± 0.2	< 7	n.d.
Cu^65^	1 ± 0.5	1.3 ± 0.4	1.10
Zn^66^	65 ± 28	360 ± 108	5.56
Se^77^	2 ± 0.4	< 60	n.d.
Cd^111^	3 ± 1.1	< 2	n.d.

Data presented as dry weight normalised shoot ionomics data obtained by inductively coupled plasma optical emission spectroscopy (ICP-OES) as per [19,20] on 5 week old Col-0 shoots grown in soil (the PiiMS database: soil-grown plants fed ½-strength MS media; n=125 plants) and our hydroponics system (n=12 plants). Ratio compares ionome of hydroponics plants to soil-grown plants showing lines are similar in nutrient content for most elements, excluding Zinc, in the shoot. Detection limit shown for Cr, Co, Ni, Se and Cd, n.d.: not determined as one or both readings were given as a detection limit (note in all these cases the ranges overlap).

The ability to isolate the entire root and shoot tissues of plants also enabled quantification of the transcriptional response to altered elemental concentrations in the growth media. We adjusted the calcium ion activity (*a*_Ca2+_) to 3 levels; 1 mM (BNS), 0.025 mM (Low Calcium Solution, LCS) and 5 mM (High Calcium Solution, HCS) (Additional file 1), whilst keeping the activity of all other ions (except Cl^–^) similar. We quantified the transcriptional response of roots and shoots to these solution changes within the epidermal enhancer trap line, KC464 (Columbia-0 background) of: known tonoplast Ca^2+^/H^+^ exchangers (*AtCAX1*, *AtCAX2*) and endoplasmic reticulum-localised autoinhibited Ca^2+^-ATPase (*AtACA2*) calcium transporter; and vacuolar H^+^-ATP synthase subunits (*AtVHA-a2, AtVHA-a3*) (Figure 2).

**Figure 3:**
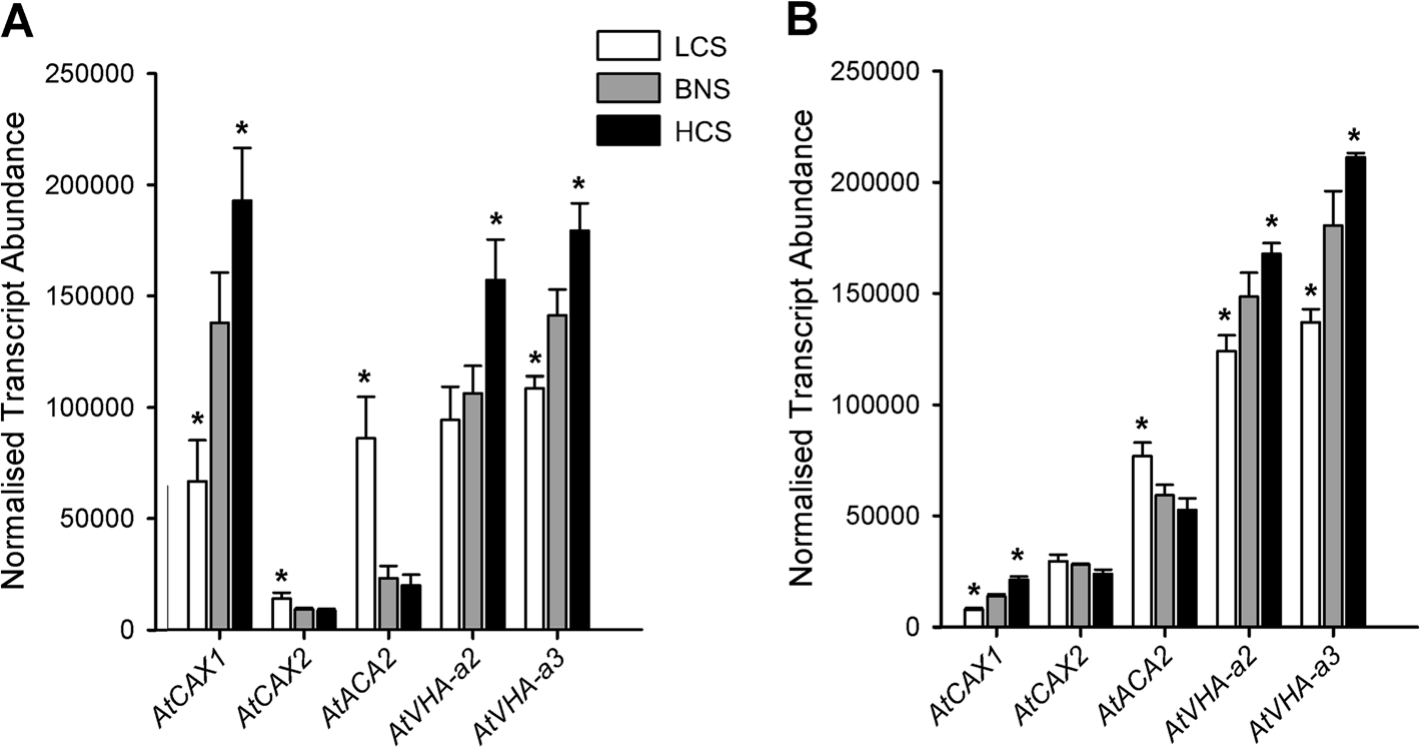
**Calcium-dependent transcriptional responses of Arabidopsis leaves and roots of hydroponically-grown plants.** qPCR performed on RNA isolated from the (**A**) shoots and (**B**) roots (above and below the hypocotyls, respectively) of 5-week old Arabidopsis KC464 plants grown under three different Ca activities (a_Ca_ LCS = 0.025mM; a_Ca_ BNS = 1 mM; a_Ca_ HCS = 5 mM) for seven days. n = 9, from three biological replicates per tissue. Mean + SD. Asterisk indicates significant expression difference from BNS (P < 0.01). qPCR performed as described in Conn *et al. *[19,20] with primers listed in Additional file 3.

We found that the expression levels of genes matched previous reports, including the higher shoot expression of *AtCAX1* and the higher root expression of both *AtCAX2* and *AtACA2* (Figure 3, Additional file 3) [26,27]. We also confirm the calcium concentration-dependent response of *AtCAX1* seen in previous reports [19,20,28]. Whilst the transcript abundance of *AtACA2* has previously been shown to be unchanged with increased Ca [26,27], we detected that its expression was increased in both shoot and root tissues in LCS. *AtACA2* and *AtCAX2* also showed the opposite transcriptional regulation to *AtCAX1* by LCS (Figure 3A,B). It is conceivable that this may be the result of a greater affinity but lower capacity for Ca ion (Ca^2+^) transport by *AtACA2* than *AtCAX1 *[29,30], so altering the capacity of the vacuole for Ca storage and increasing the role of the ER in this process over the vacuole when Ca is limiting. Likewise, the lower affinity Ca^2+^-transport capacity of AtCAX2 compared to AtCAX1 [29,30] may also contribute to the lower Ca storage of the vacuole under these conditions [19-21]. The fact that both genes are preferentially expressed in the mesophyll, adds further evidence to suggest that *AtACA2* and *AtCAX2* may play a minor role in leaf Ca compartmentation, as this is where the majority of Ca is stored [19-21,29]. In addition both genes are transcriptionally upregulated in the double knockout of two vacuolar CAX genes (*cax1/cax3*), therefore *AtACA2* and *AtCAX2* have both been predicted to partially compensate for the loss of the major mechanism to secrete leaf apoplastic Ca [19,21]. The vacuolar ATPase subunits, *AtVHA-a2* and *AtVHA-a3*, were found to show a similar Ca-dependent transcriptional response as *AtCAX1*, which is consistent with the T-DNA insertional mutant of these genes showing similar dwarf growth and lower leaf Ca accumulation phenotypes to the *cax1/cax3* line [19,31]. As such, these results validated the use of these solutions in this hydroponics system.

### Gas exchange measurement of hydroponically grown plants

**Figure 4:**
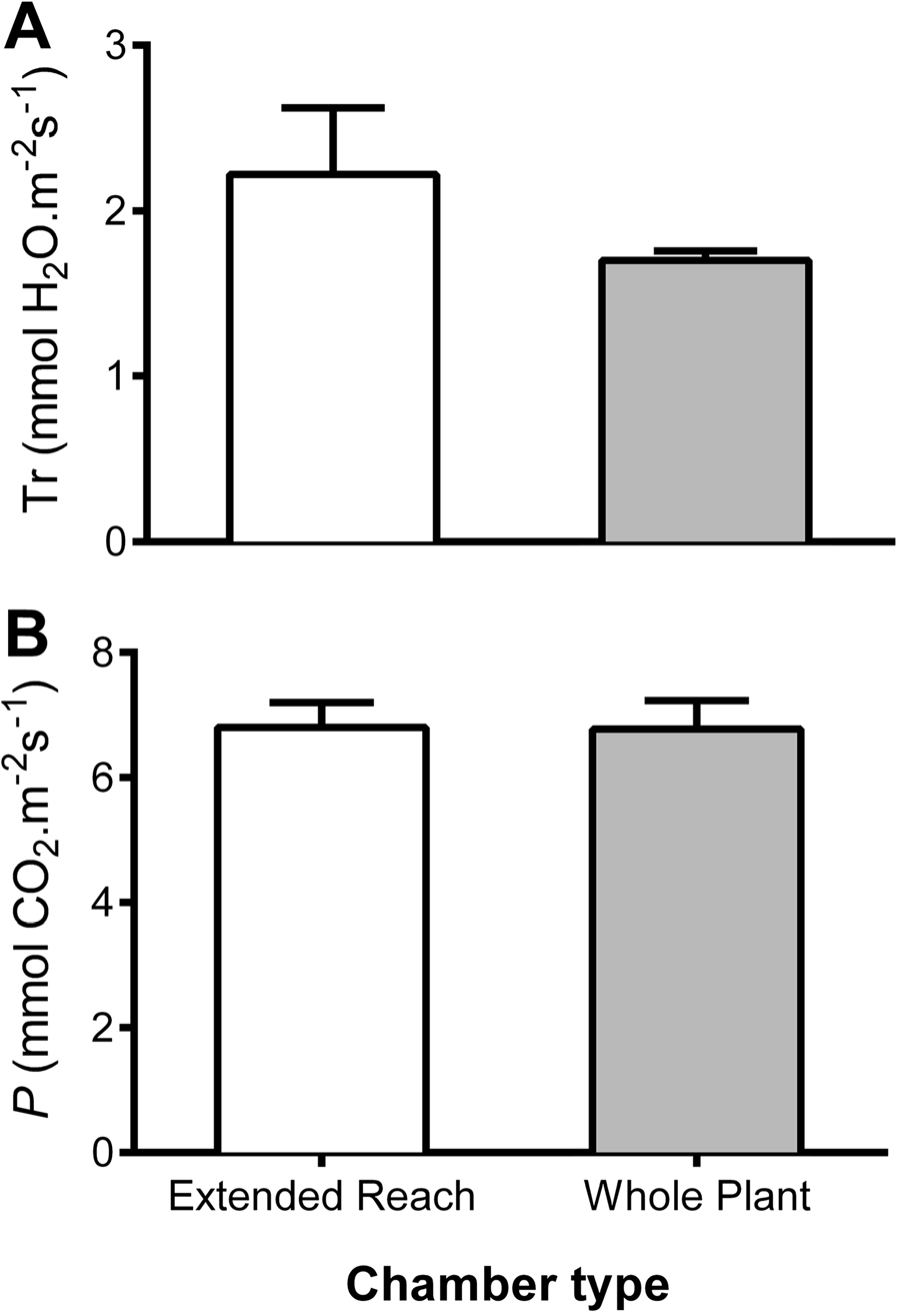
**Gas exchange measurements for Arabidopsis Col-0 measured using the LiCOR extended reach chamber or whole plant chamber whilst growing in hydroponics. A** Transpiration or **B** Net CO_2_ assimilation/photosynthesis measured using 6-week old plants growing the basal nutrient solution. Individual plants were exposed to light intensity of ~350 μmol m^-2^ s^-1^ at least 30 min prior to the start of measurement. The rosette was allowed to acclimatise inside the Arabidopsis whole rosette or extended reach chamber for at least 10 min before gas exchange data were recorded with reference CO_2_ concentration set at 500 μmol mol^-1^, flow rate at 500 μmol s^-1^ (for the whole plant chamber) or 100 μmol s^-1^ (for the extended reach chamber) light intensity at 350 μmol photons m^-2^ s^-1^ and relative humidity at 56%. Data shown as Mean + SEM of fifteen biological replicates. No significant differences were found between each dataset using a *t*-test (P < 0.01).

Measurement of gas exchange for Arabidopsis can be problematic due to the plants’ small stature and rosette growth. However, Arabidopsis can be induced to produce a relatively large amount of shoot vegetative biomass in low light conditions (~100 μmol photons.m^-2^s^-1^), and when the photoperiod is short (~8-10 h). Leaves of hydroponically grown Arabidopsis plants are relatively clean, compared to soil grown leaves, hence there is no need to wipe the leaves prior to measurement of gas exchange. This avoids any potential mechanical damage to the leaves or the trichomes, which would affect airflow and the extent of the boundary layer across the leaf, which can influence the results of gas exchange measurements. We used a LiCOR 4600-XT InfraRed Gas Analyser (IRGA), with the whole Arabidopsis chamber or extended reach chamber, to take gas exchange measurements as described in the Figure 4 legend and Additional file 2.

To be able to perform these measurements we found it necessary to make all components of the Arabidopsis whole plant chamber airtight – without this, moisture from the hydroponics media compromised the gas exchange measurements. As detailed in Additional file 2, we sealed the plant holding lid into the centrifuge tube lid using teflon air-tight sealing tape. The plant, now held within a centrifuge tube lid, was transferred into an intact centrifuge tube base containing the treatment solution of interest. The centrifuge tube was then sealed into the LiCOR ‘cone-tainer’ using a 30 mm OD rubber O-ring. This system would allow exclusive measurement of rosette gas exchange for at least 3 h for 6-week old Arabidopsis plants without any detectable reduction in photosynthetic rate during the middle of the photoperiod (Figure 4). Gas exchange measurements were adjusted on the basis of the leaf area contained within: i) the extended reach chamber (LiCOR) estimated by taking a scaled photograph and analysis of the percentage of the leaf within the chamber window using ImageJ (National Institute of Health, NIH) as detailed in [19] or, ii) the whole Arabidopsis plant chamber (LiCOR) by estimating the rosette size using a customised code developed in MATLAB® 2010b (Mathworks Inc., Natick, MA, USA) and the Image Analysis Toolbox® to process scaled photographs semi-automatically. Two codes were used, a semi-automated and an automated code. The latter recognises by colour contrast the Arabidopsis rosette to obtain automatically the cover area. The semi-automated code was used in pictures where this contrast was not detected by the automation algorithm. In this case, a tool was developed to select a region of interest (ROI) corresponding to the rosette manually to extract the cover area. See Additional files 4, 5, 6 for further details of the code and method.

The leaf gas exchange measurements were not significantly different for the hydroponics system using either the LiCOR whole Arabidopsis plant chamber or the extended leaf chamber (Figure 4). However, it was evident that the whole plant chamber took more consistent readings presumably due to the ability to sum the reading over a larger area and avoiding the need to seal the chamber directly onto the leaf tissue, which can confound results through improper sealing and/or leaf damage. We found that consistent results could be achieved with the extended leaf chamber when leaves were large enough, but the dimensions of the leaf and petiole made the clamping of a large amount of leaf area in the chamber a challenge unless the plant was older than 6 weeks. In contrast plants could be assayed in the whole Arabidopsis chamber from weeks 3-8. It is clear that this system offers potential to be widely used to study leaf gas exchange in a highly controlled manner throughout the majority of Arabidopsis development.

### Comments

#### In our hands

Given the importance of aeration of hydroponics systems for adequate growth [10], several aeration systems were trialled. The media within the tank was aerated either using a standard 4-outlet aquarium pump that constantly bubbled air through airstones or by using an ebb-and-flow system that pumped media between the tank containing the plants and a solution holding reservoir every 60 min. Both systems produced plants that at least qualitatively resembled each other, however, the former technique was markedly simpler to construct and maintain so it was used for all further studies. Oxygenation levels in the constantly aerated plants were sufficient to avoid increased expression of known hypoxia induced genes, *AtWRKY40* and *AtNIP2;1*[32,33], in contrast, transcription of both genes were induced when the media was non-aerated for 7 days (Additional file 7).

### Profiling of transgenic plants

The desire to accelerate the analysis of transgenic Arabidopsis plants has led to the design of a number of rapid approaches for selection of transformants. The method commonly used to select transformed Arabidopsis seedlings is by spreading the seeds on suitable growth media such as soil or agar. Soil is commonly used if the selection marker gene is phosphothrinocin, whereas agar is used if the marker gene is kanamycin or hygromycin. Thereafter, the putative transformants are usually transferred into soil for seed collection. The main problem with this method is the potential damage to the fragile root systems of the selected seedlings, which consequently affects their survival rate. We demonstrate that our hydroponic system can be used as an alternative to soil growth for cultivation of transformants selected on agar plates (using phosphothrinocin, kanamycin or hygromycin) as per Harrison *et al*. [34]. Over 95% of transformants survived transfer using this method, with the collected seed displaying a high germination rate (Additional file 8). However, the real advantage lies in the ability to reliably analyse mature first generation transformants, particularly for root cellular localisation studies and root phenotypes that are impossible with soil- and agar plate- based selection methods.

### Adapting the system for other plants

The improved hydroponic system we highlight here can be easily adapted for use with other plants with changes to the diameter of the hole produced in the lid of the microcentrifuge tube. We made holes of up to 6 mm in diameter (suitable for up to 4-week old cucumber and 6 week old tobacco [35]), and also grew *Lotus* spp. seedlings (data not shown). Furthermore, we also adapted the system for use with cereals using the 1.5 mL black microcentrifuge tubes with the bottom 7 mm cut off, this was sufficient to hold the seed, roots and shoots in place and removed the need for agar [36].

## Conclusions

We demonstrate the quality and versatility of our hydroponics system by profiling and comparing with soil-grown plants and previous hydroponics reports many parameters throughout the growth of Arabidopsis, including biomass, ionomics and transcriptomics. We present this hydroponics growth system as an adaptable system for characterising the entire Arabidopsis plant and other plants by a variety of physiological and molecular biological methods, superior to and more inexpensive than many techniques currently in use.

## Abbreviations

SiCSA: Single cell sampling and analysis; qPCR: Quantitative real-time RT-PCR; GFP: Green fluorescent protein; GSP: Gene-specific primer; UAS: Upstream activation sequence; Ca: Calcium; K: Potassium; Mg: Magnesium; Na: Sodium; aCa: Calcium activity; GM: Germination medium; BNS: Basal nutrient solution; LCS: Low calcium solution; HCS: High calcium solution; CAX: Calcium proton exchanger.

## Competing interest

The authors declare that they have no competing interests.

## Authors’ contributions

SC and MG designed the hydroponics system. SC, MD and MG drafted the manuscript. SC undertook growth assays, SiCSA and qPCR. MG carried out the IRGA measurements. BH performed protoplast isolation and transfection. BX developed the rapid screen for selection of transformants. MS developed the pizza tray system. MG performed vMinTEQ design of growth solutions and adapted the system for use of the whole plant chamber with MD and AA. SF designed the MATLAB® code for quantifying plant rosette cover area for IRGA measurements. VC designed and tested qPCR primers. SH and LA validated the system for imaging promoter GUS fusions and gene-GFP assays. BH, AA, BX and MG made the video. All authors read and approved the final manuscript.

## Supplementary Material

Additional file 1 Growth solutions used in this manuscript. Click here for file

Additional file 2 Detailed flowchart of plant preparation for gas exchange measurements. Click here for file

Additional file 3 qPCR primers used in this manuscript. Click here for file

Additional file 4 Details on the code developed for MATLAB® for estimating Arabidopsis rosette size. Click here for file

Additional file 5 Code developed in MATLAB used to estimate Arabidopsis rosette size. Click here for file

Additional file 6 Excel file with extra columns needed to alter LiCOR gas exchange measurements for different size rosettes. Click here for file

Additional file 7 Aerated hydroponics produce plants with no apparent oxygen transcriptional stress response. Click here for file

Additional file 8 Flow-chart and images of post-selection growth of Arabidopsis transformants. Click here for file

## References

[B1] Initiative TAG Analysis of the genome sequence of the flowering plant *Arabidopsis thaliana* Nature 2000 408 796 815 10.1038/35048692 11130711

[B2] Rhee SY Bioinformatic resources, challenges, and opportunities using Arabidopsis as a model organism in a post-genomic era Plant Physiol 2000 124 1460 1464 10.1104/pp.124.4.1460 11115859 PMC1539296

[B3] Hershey DR Solution culture hydroponics: history & inexpensive equipment Am. Biol. Teacher 1994 56 111 118 10.2307/4449764

[B4] Jones JB Jr Hydroponics: Its history and use in plant nutrition studies J 1982 5 1003 1030 10.1080/01904168209363035

[B5] Ahn SJ Shin R Schachtman DP Expression of *KT/KUP* genes in Arabidopsis and the role of root hairs in K^+^ uptake Plant Physiol 2004 134 1135 1145 10.1104/pp.103.034660 14988478 PMC389937

[B6] Araponics: hydroponic growing system for Arabidopsis thaliana 2010 http://www.araponics.com/

[B7] Robison MM Smid MPL Wolyn DJ High-quality and homogeneous *Arabidopsis thaliana* plants from a simple and inexpensive method of hydroponic cultivation Can J Bot 2006 84 1009 1012 10.1139/b06-054

[B8] Arteca RN Arteca JM A novel method for growing *Arabidopsis thaliana* plants hydroponically Physiol Plant 2000 108 188 193 10.1034/j.1399-3054.2000.108002188.x

[B9] Schlesier B Bréton F Mock H-P A hydroponic culture system for growing *Arabidopsis thaliana* plantlets under sterile conditions Plant Mol Biol Rep 2003 21 449 456 10.1007/BF02772594

[B10] Smeets K Ruytinx J Van Belleghem F Semane B Lin D Vangronsveld J Cuypers A Critical evaluation and statistical validation of a hydroponic culture system for *Arabidopsis thaliana* Plant Physiol Biochem 2008 46 212 218 10.1016/j.plaphy.2007.09.014 18024051

[B11] Huttner D Bar-Zvi D An improved, simple, hydroponic method for growing *Arabidopsis thaliana* Plant Mol Biol Rep 2003 21 59 63 10.1007/BF02773397

[B12] Gibeaut DM Hulett J Cramer GR Seemann JR Maximal biomass of *Arabidopsis thaliana* using a simple, low-maintenance hydroponic method and favorable environmental conditions Plant Physiol 1997 115 317 319 10.1104/pp.115.2.317 9342857 PMC158488

[B13] Tocquin P Corbesier L Havelange A Pieltain A Kurtem E Bernier G Périlleux C A novel high efficiency, low maintenance, hydroponic system for synchronous growth and flowering of *Arabidopsis thaliana* BMC Plant Biol 2003 3 2 10.1186/1471-2229-3-2 12556248 PMC150571

[B14] Berezin I Elazar M Gaash R Avramov-Mor M Shaul O Asao T The use of hydroponic growth systems to study the root and shoot ionome of *Arabidopsis thaliana* Hydroponics - A Standard Methodology for Plant Biological Researches 2012 InTech

[B15] Hermans C Verbruggen N Physiological characterization of Mg deficiency in *Arabidopsis thaliana* J Exp Bot 2005 56 2153 2161 10.1093/jxb/eri215 15983014

[B16] Coosemans J Control of algae in hydroponic systems ISHS Acta Hort 1995 382 263 268

[B17] Boller T Felix G A renaissance of elicitors: perception of microbe-associated molecular patterns and danger signals by pattern-recognition receptors Annu Rev Plant Biol 2009 60 379 406 10.1146/annurev.arplant.57.032905.105346 19400727

[B18] Huang C Verrillo F Renzone G Arena S Rocco M Scaloni A Marra M Response to biotic and oxidative stress in ***Arabidopsis thaliana: *****analysis of variably phosphorylated proteins** J Proteomics 2011 74 1934 1949 10.1016/j.jprot.2011.05.016 21619950

[B19] Conn SJ Gilliham M Athman A Schreiber AW Baumann U Moller I Cheng N-H Stancombe MA Hirschi KD Webb AAR Burton R Kaiser BN Tyerman SD Leigh RA Cell-specific vacuolar calcium storage mediated by *CAX1* regulates apoplastic calcium concentration, gas exchange, and plant productivity in Arabidopsis Plant Cell 2011 23 240 257 10.1105/tpc.109.072769 21258004 PMC3051233

[B20] Conn SJ Conn V Tyerman SD Kaiser BN Leigh RA Gilliham M Magnesium transporters, MGT2/MRS2-1 and MGT3/MRS2-5, are important for magnesium partitioning within *Arabidopsis thaliana* mesophyll vacuoles New Phytol 2011 190 583 594 10.1111/j.1469-8137.2010.03619.x 21261624

[B21] Gilliham M Athman A Tyerman SD Conn SJ Cell-specific compartmentation of mineral nutrients is an essential mechanism for optimal plant productivity–another role for TPC1? Plant Signal Behav 2011 6 1656 1661 10.4161/psb.6.11.17797 22067997 PMC3329329

[B22] Roy SJ Conn SJ Mayo GM Athman A Gilliham M Shabala S, Cuin TA Transcriptomics on small samples Methods in Molecular Biology: Plant Salt Tolerance 2012 New Jersey, USA: Humana Press

[B23] Mündermann L Erasmus Y Lane B Coen E Prusinkiewicz P Quantitative modeling of Arabidopsis development Plant Physiol 2005 139 960 968 10.1104/pp.105.060483 16183852 PMC1256009

[B24] Cheng N-H Pittman JK Shigaki T Lachmansingh J LeClere S Lahner B Salt DE Hirschi KD Functional association of Arabidopsis CAX1 and CAX3 is required for normal growth and ion homeostasis Plant Physiol 2005 138 2048 2060 10.1104/pp.105.061218 16055687 PMC1183394

[B25] Yoo S-D Cho Y-H Sheen J Arabidopsis mesophyll protoplasts: a versatile cell system for transient gene expression analysis Nat Protocols 2007 2 1565 1572 10.1038/nprot.2007.199 17585298

[B26] Hirschi KD Expression of Arabidopsis *CAX1* in tobacco: altered calcium homeostasis and increased stress sensitivity Plant Cell 1999 11 2113 2122 10559438 10.1105/tpc.11.11.2113 PMC144126

[B27] Edmond C Shigaki T Ewert S Nelson MD Connorton JM Chalova V Noordally Z Pittman JK Comparative analysis of CAX2-like cation transporters indicates functional and regulatory diversity Biochem J 2009 418 145 154 10.1042/BJ20081814 18950291

[B28] Maathuis FJM Filatov V Herzyk P Krijger G Axelsen K Chen S Green BJ Li Y Madagan KL Sánchez-Fernández R Forde BG Palmgren MG Rea PA Williams LE Sanders D Amtmann A Transcriptome analysis of root transporters reveals participation of multiple gene families in the response to cation stress Plant J 2003 35 675 692 10.1046/j.1365-313X.2003.01839.x 12969422

[B29] Conn S Gilliham M Comparative physiology of elemental distributions in plants Ann Bot 2010 105 1081 1102 10.1093/aob/mcq027 20410048 PMC2887064

[B30] Korenkov V Hirschi K Crutchfield JD Wagner GJ Enhancing tonoplast Cd/H antiport activity increases Cd, Zn, and Mn tolerance, and impacts root/shoot Cd partitioning in *Nicotiana tabacum* L Planta 2007 226 1379 1387 10.1007/s00425-007-0577-0 17636324

[B31] Krebs M Beyhl D Görlich E Al-Rasheid KA Marten I Stierhof YD Hedrich R Schumacher K Arabidopsis V-ATPase activity at the tonoplast is required for efficient nutrient storage but not for sodium accumulation Proc Natl Acad Sci USA 2010 107 3251 3256 10.1073/pnas.0913035107 20133698 PMC2840351

[B32] Liu F Vantoai T Moy LP Bock G Linford LD Quackenbush J Global transcription profiling reveals comprehensive insights into hypoxic response in Arabidopsis Plant Physiol 2005 137 1115 1129 10.1104/pp.104.055475 15734912 PMC1065411

[B33] Vlad F Spano T Vlad D Daher FB Ouelhadj A Fragkostefanakis S Kalaitzis P Involvement of Arabidopsis prolyl 4 hydroxylases in hypoxia, anoxia and mechanical wounding Plant Signal Behav 2007 2 368 369 10.4161/psb.2.5.4462 19704601 PMC2634214

[B34] Harrison SJ Mott EK Parsley K Aspinall S Gray JC Cottage A A rapid and robust method of identifying transformed *Arabidopsis thaliana* seedlings following floral dip transformation Plant Methods 2006 2 19 10.1186/1746-4811-2-19 17087829 PMC1636043

[B35] Casal JJ Ballare CL Tourn M Sanchez RA Anatomy, growth and survival of a long-hypocotyl mutant of *Cucumus sativus* deficient in phytochrome B Ann Bot 2012 73 569 575

[B36] Munns R James RA Xu B Athman A Conn SJ Jordans C Byrt CS Hare RA Tyerman SD Tester M Plett D Gilliham M Wheat grain yield on saline soils is improved by an ancestral Na^+^ transporter gene Nat Biotech 2012 30 360 364 10.1038/nbt.2120 22407351

[B37] Nakagawa T Suzuki T Murata S Nakamura S Hino T Maeo K Tabata R Kawai T Tanaka K Niwa Y Watanabe Y Nakamura K Kimura T Ishiguro S Improved Gateway binary vectors: high-performance vectors for creation of fusion constructs in transgenic analysis of plants Biosci Biotechnol Biochem 2007 71 2095 2100 10.1271/bbb.70216 17690442

