# Supplementary material for: Protocol: optimising hydroponic growth systems for nutritional and physiological analysis of Arabidopsis thaliana and other plants
Source: Plant Methods. 2013 Feb 5;9:4. doi: 10.1186/1746-4811-9-4 (PMC3610267; doi:10.1186/1746-4811-9-4)
Supplement: Additional file 2 — Detailed flowchart of plant preparation for gas exchange measurements. [file 1746-4811-9-4-S2.doc]

**Additional File 2 – Detailed flowchart of plant preparation for gas exchange measurements. ***GM = germination medium.

**
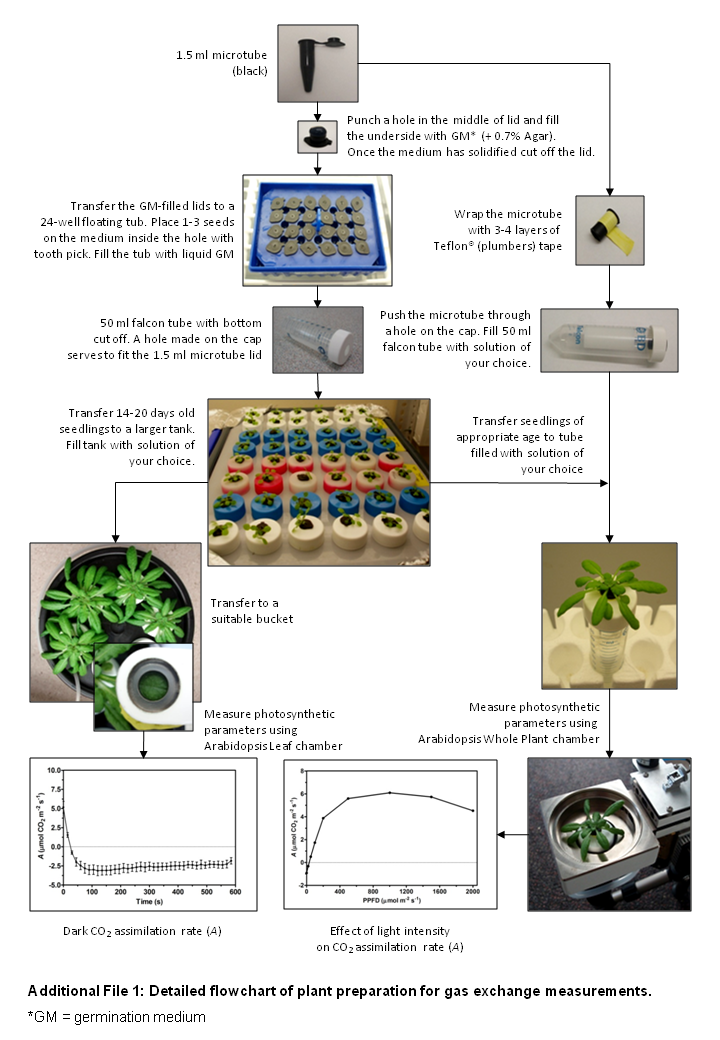
**
