# Supplementary material for: Protocol: optimising hydroponic growth systems for nutritional and physiological analysis of Arabidopsis thaliana and other plants
Source: Plant Methods. 2013 Feb 5;9:4. doi: 10.1186/1746-4811-9-4 (PMC3610267; doi:10.1186/1746-4811-9-4)
Supplement: Additional file 3 — qPCR primers used in this manuscript. [file 1746-4811-9-4-S3.doc]

**Additional File 3: Nested and quantitative PCR primer sequences used in this study.**

qF: qPCR forward primer, qR: qPCR reverse primer (used for qPCR or 2^nd^ round of SiCSA nested PCR). N: all 4 dNTPs. Annotated those primers used for normalisation in all qPCR experiments.

| **Gene Name** | **ATG Identifier** | **Primer Sequence (5’ → 3’)** |
| --- | --- | --- |
| **Quantitative PCR primers** | | |
| *AtActin2* (qPCR normalisation) | At3g18780 | qF: TGAGCAAAGAAATCACAGCACT  qR: CCTGGACCTGCCTCATCATAC |
| *AtCyclophilin* (qPCR normalisation) | At2g36130 | qF: TGGCGAACGCTGGTCCTAATACA  qR: CAAAAACTCCTCTGCCCCAATCAA |
| *AtEF-1α* (qPCR normalisation) | At1g07940 | qF: GACAGGCGTTCTGGTAAGGAG  qR: GCGGAAAGAGTTTTGATGTTCA |
| *AtGAPDH-A* (qPCR normalisation) | At3g26650 | qF: TGGTTGATCTCGTTGTGCAGGTCTC  qR: GTCAGCCAAGTCAACAACTCTCTG |
| *Atβ-Tubulin 5* (qPCR normalisation) | At1g20010 | qF: CGTGAAATCCAGCGTTTGTGA  qR: TCGTCCATTCCTTCTCCTGTG |
| *AtWRKY40* | At1g80840 | qF: AAATCAGCCCTCCCAAGAAACG  qR: CTTCACGACAGTCTCTTCTCTCTGC |
| *AtVHA-a2* | At2g21410 | qF: CATATTTGCGACTGTGGGAGTGC  qR: ACTTGTAACCATCGCCTTCG |
| *AtVHA-a3* | At4g39080 | qF: TTTGCTGGTGATGGAGACAC  qR: TGCCGTGAAAATGAAAGTGA |
| *AtCAX1* | At2g38170 | qF: CATCATCGTGGCGTGGATT  qR: GCATTTTGTTTCTGGGGAAGT |
| *AtCAX2* | At3g13320 | qF: TTCCATGTTTGCGGTCCC  qR: CCCTTTTATGCTTCACACCAGA |
| *AtACA2* | At4g37640 | qF: AAGGGAAGGCTATGTTTGGTCT  qR: TGTGTTATTGTGAGTGGTGTTGTG |
