# Supplementary material for: Protocol: optimising hydroponic growth systems for nutritional and physiological analysis of Arabidopsis thaliana and other plants
Source: Plant Methods. 2013 Feb 5;9:4. doi: 10.1186/1746-4811-9-4 (PMC3610267; doi:10.1186/1746-4811-9-4)
Supplement: Additional file 4 — Details on the code developed for MATLAB® for estimating Arabidopsis rosette size. [file 1746-4811-9-4-S4.doc]

**Additional File 4 – Description of how to use the rosette size estimation tool.**

To estimate the rosette size of Arabidopsis or other plants using the whole Arabidopsis chamber a customised code developed in MATLAB® 2010b (Mathworks Inc., Natick, MA, USA) and the Image Analysis Toolbox® was used to process scaled photographs semi-automatically. Two codes were used, a semi-automated and an automated code. The latter recognises by colour contrast the Arabidopsis rosette to obtain automatically the cover area. The semi-automated code was used in pictures where this contras**t** was not detected by the automation algorithm. In this case, a tool was developed to select a region of interest (ROI) corresponding to the rosette manually to extract the cover area.

To estimate the size of the rosette do the following:

1. Take a series of photographs saved in the jpeg format. Ensure in each photograph you have a black square of 10 x 10 mm placed outside the perimeter of the Arabidopsis rosette. Also use white paper either side of the rosette to provide adequate contrast.


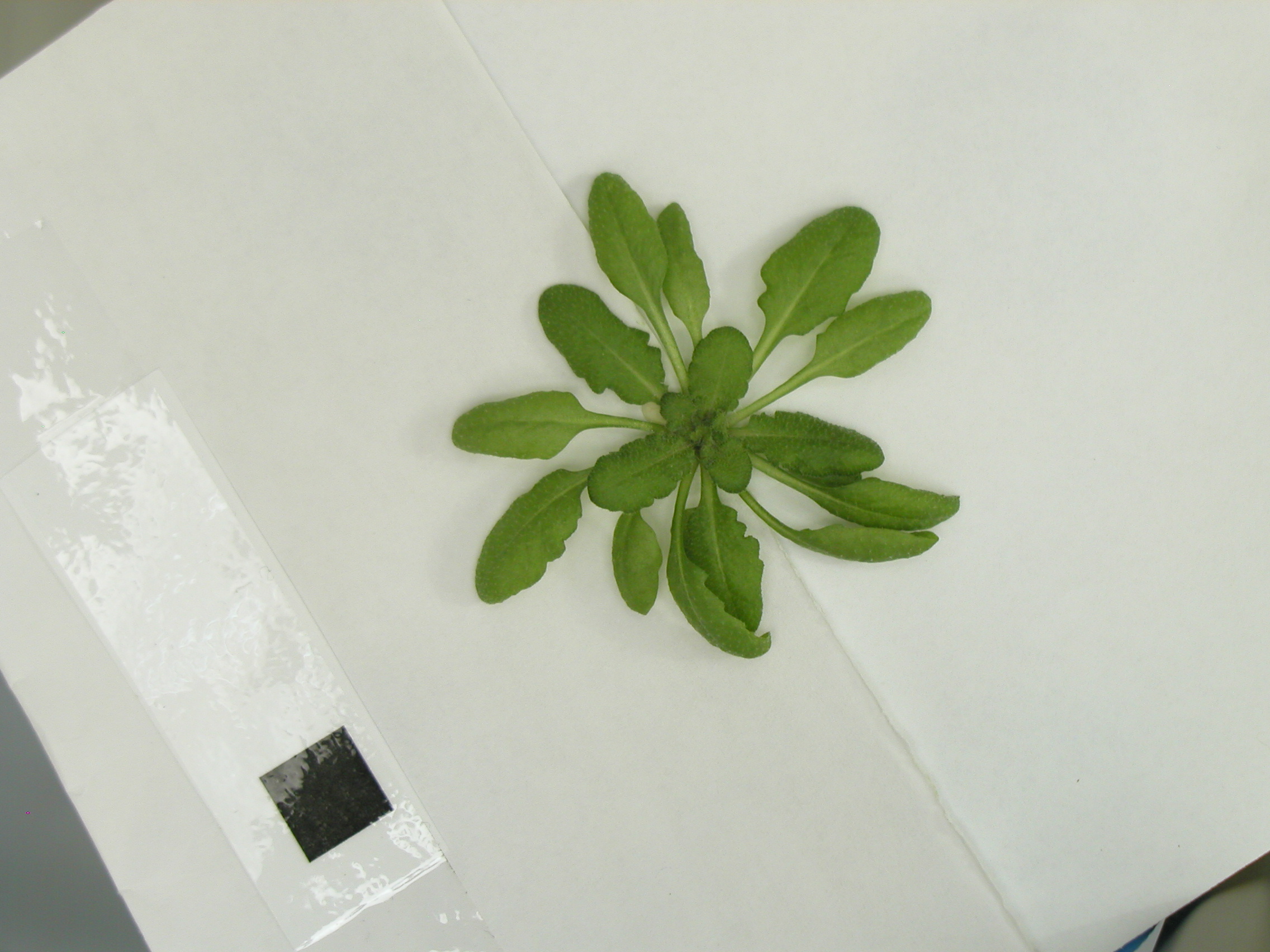


1. Open MATLAB® 2010b.
2. Copy the Leaf Area Index code from Additional File 7 into MATLAB editor. Save into a new working directory as LAI.m
3. Copy photographs to the same directory, re-label the photographs numerically (i.e 1-XX)
4. Run LAI.m
5. Enter initial pic number:1
6. Enter ending pic number: XX
7. Drag and drop to capture the 10mm X 10mm black square, then double click inside selected area
8. Click between the two peaks of the histogram
9. Count the number of significant overlapping leaf areas of the rosette
10. Drag and drop to capture the rosette, then double click inside selected area
11. Click between the two peaks of the histogram
12. Input number of rosettes: XX (i.e. input the count of overlapping leaf areas from step 10)
13. Select each overlapping leaf area using mouse pointer, and then double click inside selected area.
14. Repeat steps 8-14 for each photograph. Matlab will output excel file (LAI.xls) into the directory. The first column indicates raw leaf area (cm^2^); the second column indicates total leaf area (corrected for leaf overlap). Units are in cm^2^.
15. Download the excel data file from the LiCOR device following manufacturer’s instructions
16. Copy and paste last five columns in Additional File 8 to the end of the LiCOR output file.
17. Copy new area for each rosette (from second column of LAI.xls) and outputs will be given for area adjusted photosynthesis and transpiration.
