# Supplementary material for: Protocol: optimising hydroponic growth systems for nutritional and physiological analysis of Arabidopsis thaliana and other plants
Source: Plant Methods. 2013 Feb 5;9:4. doi: 10.1186/1746-4811-9-4 (PMC3610267; doi:10.1186/1746-4811-9-4)
Supplement: Additional file 5 — Code developed in MATLAB used to estimate Arabidopsis rosette size. [file 1746-4811-9-4-S5.doc]

**Additional File 5 - Code developed in MATLAB used to estimate Arabidopsis rosette size**

%---------Script to calculate leaf area and leaf morphological indices-----

%---------------------------from digital images----------------------------

% First calculates all plant area

clear all

f0 = 'XXX.JPG';

%Call values for image files with JPG extension and name DSCN and variables

%for sub-image analysis

firstpic = input('Enter initial pic number :');

lastpic= input('Enter ending pic number :');

%Reference area extraction

%Loops to generate binary images for area calculation

for i=firstpic:lastpic;

filename = strrep(f0,'XXX',num2str(i));

im = imread(filename);

I=imcrop(im);warning off;

close(1);

layer_blue=I(:,:,3);

%Separates Blue Layer from image

hist_blue=imhist(layer_blue);

plot(hist_blue);

title(['Blue Histogram from ',filename]);

%Uses the mouse pointer to input T (x-axis)

hold on;

datacursormode on

[T,y]=ginput(1);

close(1);

bw = I(:,:,3)>T;

Ar=bwarea(~bw);

Aref=Ar/1;

%Call image a second time for plant area measurement

I2=imcrop(im);warning off;

close(1);

layer_blue=I2(:,:,3);

%Separates Blue Layer from image

hist_blue=imhist(layer_blue);

plot(hist_blue);

title(['Blue Histogram from ',filename]);

%Uses the mouse pointer to input T (x-axis)

hold on;

datacursormode on

[T1,y]=ginput(1);

close(1);

bw2 = I2(:,:,3)>T1;

A1=bwarea(~bw2);

%Select internal overlapping rosette

ROS=input('Input number of rossetes:');

if ROS <= 0

AS1 = 0;

else

for k=1:ROS

A=roipoly(I2);

AS1(k,1)=bwarea(A);

end

end

ROS2=sum(AS1);

%Total area = Main area + internal rossetes

TA(i,1)=(A1+ROS2)/Aref;

CA(i,1)=A1/Aref;

end

y=TA>0; %Filtering images before a determine initial number

R=[CA(y) TA(y)]

Units = ('cm^2')%Results considering only cover leaf area and total area

xlswrite('LAI.xls', [R], 1, 'A1'); warning off;
