# Supplementary material for: Protocol: optimising hydroponic growth systems for nutritional and physiological analysis of Arabidopsis thaliana and other plants
Source: Plant Methods. 2013 Feb 5;9:4. doi: 10.1186/1746-4811-9-4 (PMC3610267; doi:10.1186/1746-4811-9-4)

**Additional File 7 – Aerated hydroponics produce plants with no apparent oxygen transcriptional stress response.**

qPCR performed on RNA isolated from the whole root tissue of six-week old Arabidopsis Col-0 plants grown in soil or hydroponics (BNS, a_Ca_= 1 mM). Root material obtained from plants in soil or hydroponics with and without aeration for the terminal seven days. n = 9, from three biological replicates per tissue. Mean + SD.


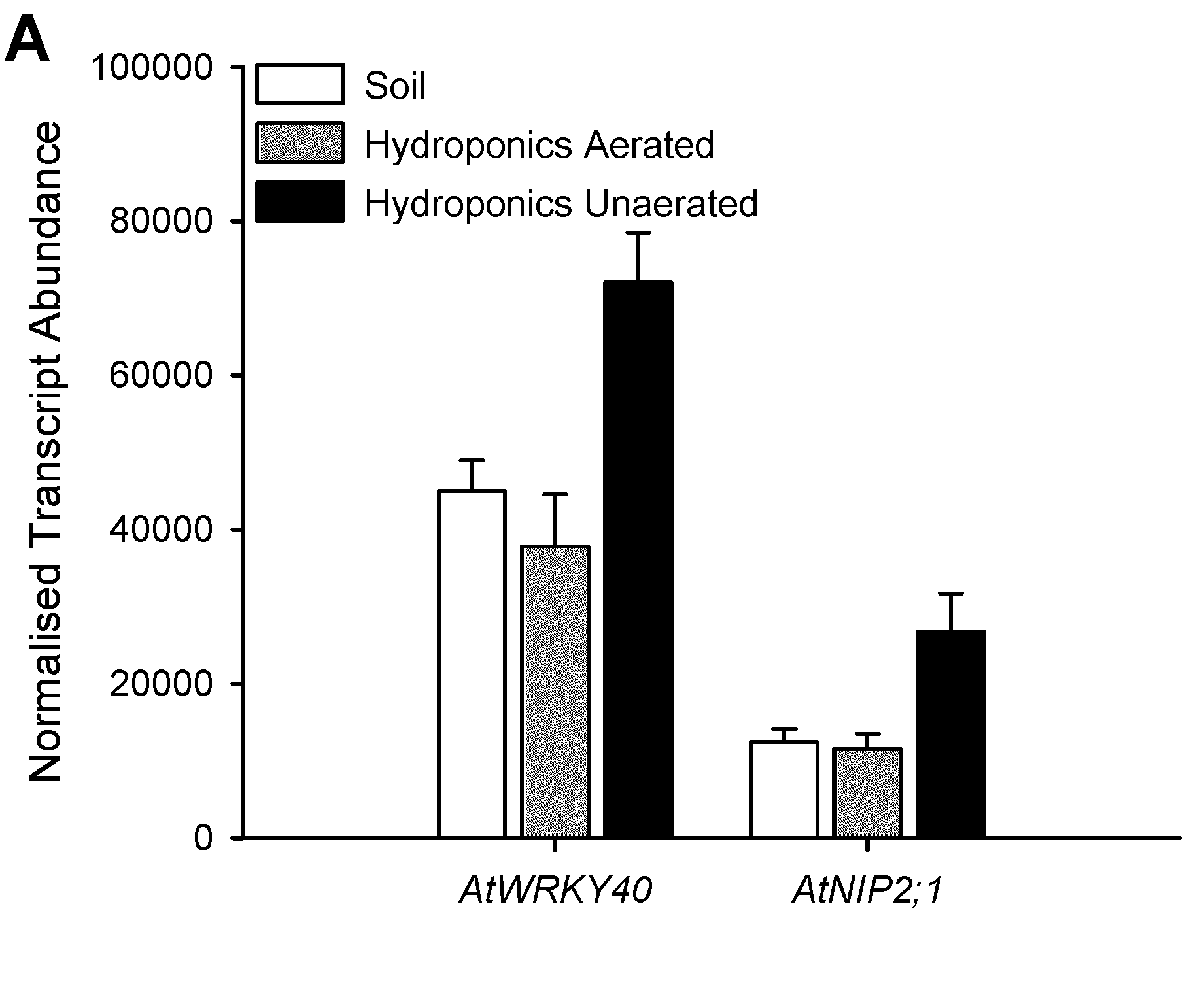

Supplement: Additional file 7 — Aerated hydroponics produce plants with no apparent oxygen transcriptional stress response. [file 1746-4811-9-4-S7.doc]
