# Supplementary material for: Protocol: optimising hydroponic growth systems for nutritional and physiological analysis of Arabidopsis thaliana and other plants
Source: Plant Methods. 2013 Feb 5;9:4. doi: 10.1186/1746-4811-9-4 (PMC3610267; doi:10.1186/1746-4811-9-4)

**Additional File 8: Flow-chart and images of post-selection growth of Arabidopsis transformants.**

Following selection of Arabidopsis transformants by Harrison *et al*. [33], seedlings as young as 3.25 days (applicable to kanamycin-, phosphinothricin- and hygromycin B-resistance) are transferred into hydroponic system. A larger hole (6 mm diameter instead of 1.5 mm) is punched into the lids of 1.5 mL black microfuge tubes. The lid is filled with BNS + 0.7% agar (note: not GM solution). Using yellow tips (20-200 μl) individual transformants are transferred from selection plate/media onto the top of agar (flow diagram). Using a supple plastic stick (10 μl tips also work) the roots are gently pushed into the BNS + 0.7% agar (gently pushing assists the roots to penetrate the agar plug). After transferring all of them into the chamber and floating them on BNS medium, the box is covered to maintain humidity and placed under short-day growth conditions (8 h:16 h photoperiod).


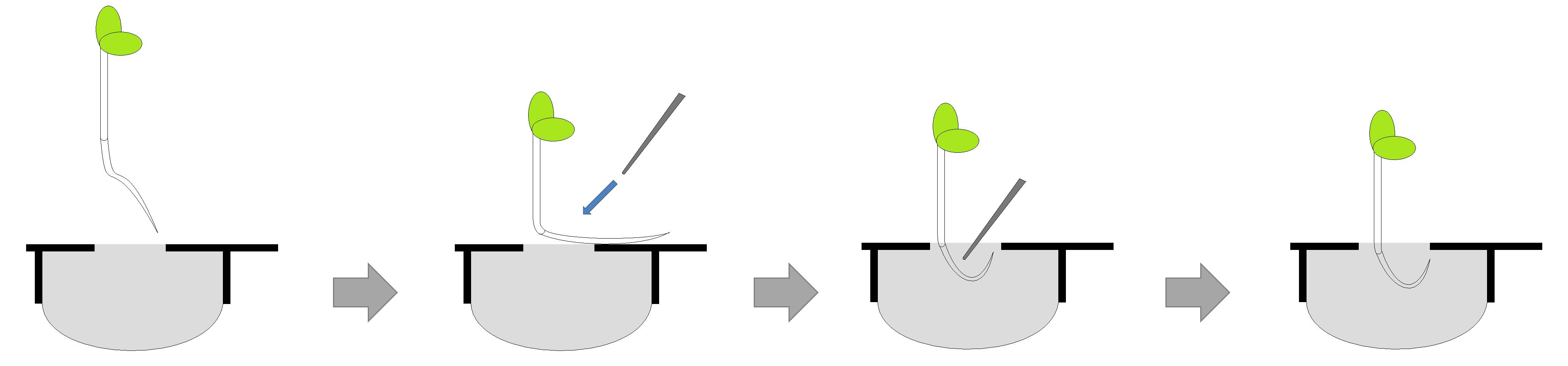


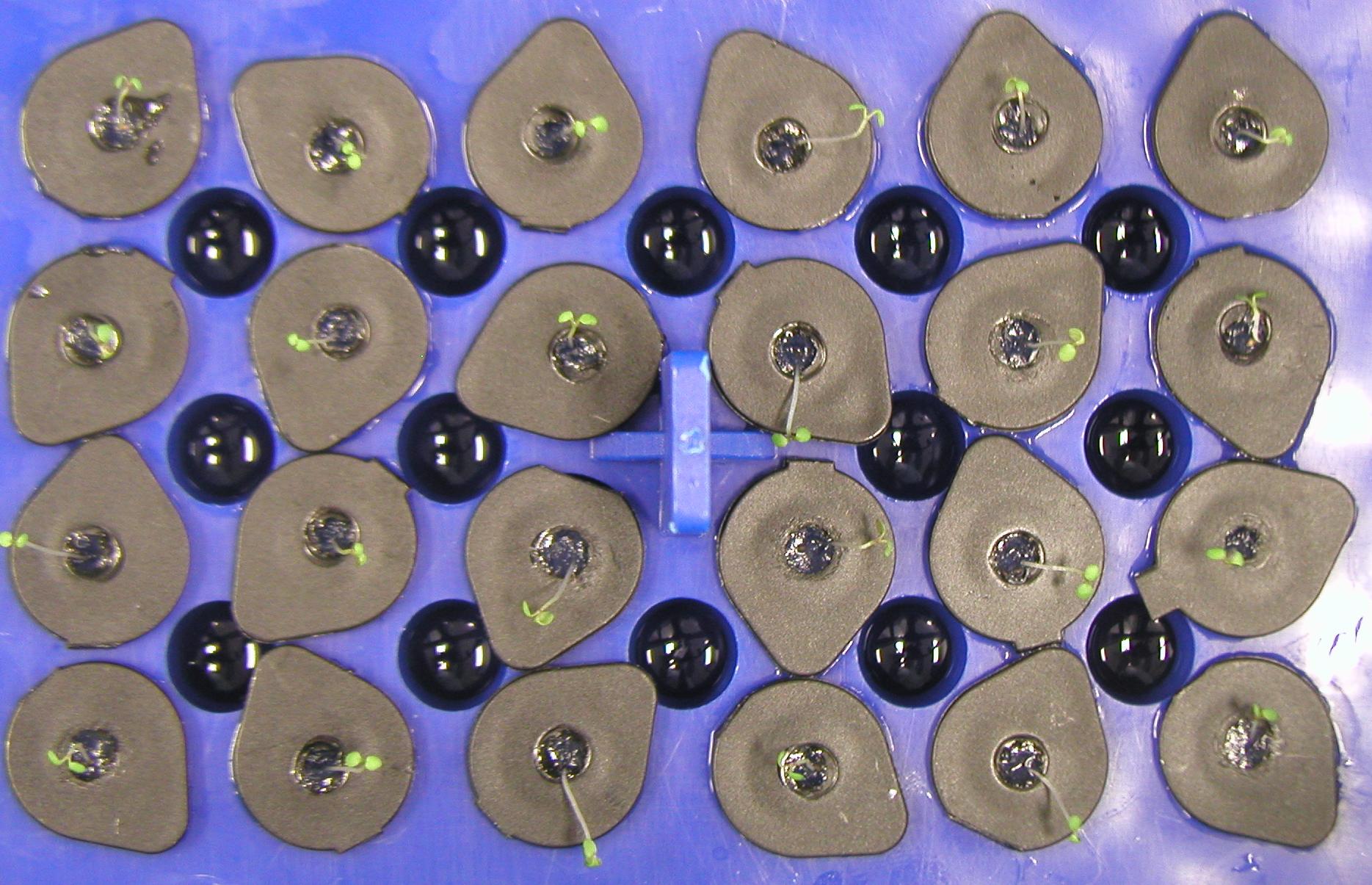

Supplement: Additional file 8 — Flow-chart and images of post-selection growth of Arabidopsis transformants. [file 1746-4811-9-4-S8.doc]
